# Endoscopic ultrasound‐guided gallbladder drainage for jaundice: Response to Vanella *et al*.

**DOI:** 10.1111/den.14886

**Published:** 2024-07-25

**Authors:** Antoine Debourdeau, Diane Lorenzo

**Affiliations:** ^1^ CHU de Nîmes Montpellier Univ MUSE, Gastroenterology Nimes France; ^2^ CHU de Montpellier Montpellier Univ MUSE, Gastroenterology Montpellier France; ^3^ Beaujon Hospital Assistance publique des Hôpitaux de Paris AP‐HP Paris France

To the Editor:

We appreciate Vanella *et al*.'s insightful letter regarding our GALLBLADEUS study.^1^ They correctly noted that endoscopic ultrasound‐guided gallbladder drainage (EUS‐GBD) may have appeared as a third‐line option. Due to our retrospective data, we lack specific details, but in our center, EUS‐GBD is often preferred over endoscopic ultrasound‐guided choledochoduodenostomy (EUS‐CDS) after failed endoscopic retrograde cholangiopancreatography, with many patients receiving EUS‐GBD as a second‐line treatment.

We fully agree with Vanella's remark that the presence of duodenal stenosis makes the use of EUS‐CDS inappropriate. However, the patients included in this study were treated at a time when this information had not yet been published, particularly by the CABRIOLET trial^2^ conducted by our correspondents. The proportion of patients with duodenal stenosis was significant but comparable in both groups (48.7% EUS‐CDS vs. 41.5% EUS‐GBD). However, despite this, our study still showed that dysfunctions seemed less frequent in the EUS‐GBD group.

Emerging evidence suggests hepaticogastrostomy as a better route for duodenal stenosis,^2^, ^3^ although it has a longer learning curve compared to EUS‐GBD, which is simpler for less‐experienced centers. Our study suggests fewer dysfunctions with EUS‐GBD vs. EUS‐CDS in this context, a finding that needs confirmation from future prospective, comparative studies as suggested by Vanella *et al*. We agree that biliary drainage far from the tumor warrants comparing EUS‐GBD to hepaticogastrostomy. The significant proportion of duodenal stenosis in our study favors EUS‐GBD, suggesting fewer dysfunctions, although this needs confirmation by future studies. This question is of interest because EUS‐GBD is simpler for less‐experienced centers and could be more widely adopted than hepaticogastrostomy. Future prospective studies comparing EUS‐CDS, EUS‐GBD, and hepaticogastrostomy across various clinical scenarios are essential. We thank Vanella *et al*. for their valuable input and look forward to further dialogue and research in this evolving field.

Authors declare no conflict of interest for this article.

## References

[1] Debourdeau A , Daniel J , Caillo L *et al*. Effectiveness of endoscopic ultrasound (EUS)‐guided choledochoduodenostomy vs. EUS‐guided gallbladder drainage for jaundice in patients with malignant distal biliary obstruction after failed endoscopic retrograde cholangiopancreatography: Retrospective, multicenter study (GALLBLADEUS study). Dig Endosc 2025; 37: 103–114. 10.1111/den.14750 PMC11718144 38380564

[2] Vanella G , Bronswijk M , Wanrooij RL *et al*. Combined endoscopic management of biliary and gastric outlet obstruction (CABRIOLET study): A multicenter retrospective analysis. DEN Open 2023; 3: e132. 35898844 10.1002/deo2.132 PMC9307724

[3] Debourdeau A , Caillol F , Zemmour C *et al*. Endoscopic management of concomitant biliary and duodenal malignant obstruction: Impact of the timing of drainage for one vs. two procedures and the modalities of biliary drainage. Endosc Ultrasound 2021; 10: 124–133. 33818527 10.4103/EUS-D-20-00159 PMC8098836

